# Toward an Etiology of Spaceflight Neuroplastic Syndrome: Evolutionary Science Leads to New Hypotheses and Program Priorities

**DOI:** 10.3390/neurosci4040021

**Published:** 2023-09-25

**Authors:** Margaret Boone Rappaport, Christopher J. Corbally

**Affiliations:** 1 The Human Sentience Project, LLC, Tucson, AZ 85704, USA; 2 Vatican Observatory, Department of Astronomy, University of Arizona, Tucson, AZ 85721, USA; corbally@as.arizona.edu

**Keywords:** neuroplasticity, spaceflight, scientific reasoning, methodology (laboratory), methodology (theoretical), Extended Evolutionary Synthesis, dopamine, serotonin, neurotrophic factor, reward system, BDNF (brain-derived neurotrophic factor), CO_2_, radiation, evolution, phenotypic adaptation, space medicine, civilian space programs, LEO (low Earth orbit), artificial gravity

## Abstract

Evolutionary theory is applied to recent neuroscientific findings on factors associated with risk-and-reward systems, and consequently, aspects of human decision making in spaceflight. Factors include enzymes aiding metabolic pathways of dopamine and serotonin; neurotrophic factors supporting neuronal functioning and plasticity; and genes associated with serotonin and dopamine systems. Not all factors are at risk in spaceflight. Some remain stable. It is hypothesized that neural deconditioning in spaceflight arises from faulty signals sent to the brain and gut in attempting to adapt phenotypically to a novel space environment. There is a mismatch between terrestrial selection pressures during human evolution and conditions of cosmic radiation, microgravity, and higher CO_2_, which together cause scattered results. A contrary question is broached: Given these findings, why are human sequelae not worse? Discussion of programmatic issues then focuses on methodologies to determine the suitability of civilians for spaceflight, an issue that grows more pressing while more varied populations prepare for spaceflight in LEO and on, and in orbit around the Moon.

## 1. Biocultural Context of Spaceflight Deconditioning: Dangers and Benefits

In the last century, researchers on human and other mammalian systems have intuitively understood the nature of neuroplasticity through keen observation. They could see it operate in young mammals, and then as the animals matured, the neuroplasticity and the ability to change and heal neurologically would largely be lost to different degrees for various species. More recently, it is theorized that very young humans are seen to be neurologically changed by aspects of their earliest environments, and the last—for example, learning a specific culture and language from a human social group [1]. As in so much of modern evolutionary theory, Dawin’s original insights [2] morphed and were further augmented to become the Extended Evolutionary Synthesis [3,4]. A perspective of conjoined biological and cultural development is emerging. The early part of the mammalian developmental cycle is far more variable and flexible than once thought and this is especially true for the higher Primates. Among animals in that biological order, neuroplasticity allows the learning of advanced neurocognitive skills through adolescence and even adulthood, and the teaching of the same into senescence.

The enormous pay-off is that the basic biology of humans has given the species the ability to operate effectively in space. Descended from an as-yet-unidentified Miocene ape [5], humans are agile, flexible, neuroplastic, and intensely social—even at long distances. In fact, they are more neuroplastic than their ape ancestors, and eventually, *Homo sapiens* evolved to be more neuroplastic than other hominins like the Neanderthals and Denisovans [1]. Neuroplasticity is an extremely beneficial feature on Earth for the cognitive development of our species, but in microgravity, with higher CO_2_ and cosmic radiation, the effects of neuroplasticity can be detrimental. The human body attempts a phenotypic adaptation to space, which appears to go awry—but only partially. Some neurological elements appear stable. The picture provided by what we call the Spaceflight Neuroplastic Syndrome in mammals is varied, almost “scattered”, according to findings from Popova and colleagues [6]. Only parts of the brain, some enzymes, some neurotrophic factors, and some genes associated with plasticity are changed. Findings from further studies in the future will explain why this variation occurs.

**Figure 1:**
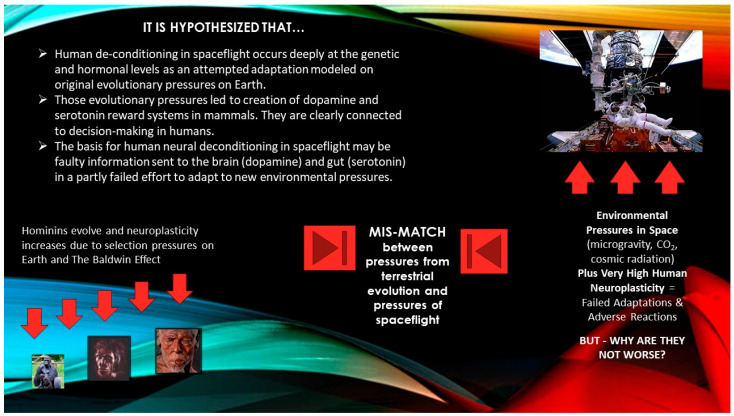
**The mismatch of evolutionary pressures and the conditions of human spaceflight.** Credits: NASA. Ape, Gorilla gorilla (**left** figure), and Homo sapiens (**right** figure), Natural History Museum, London. Homo erectus (**middle** figure), Smithsonian, Washington DC.

We hypothesize that scattered findings suggest an Earth-evolved mammal in space is attempting to adapt to conditions that do not reflect the original evolutionary selection pressures giving rise to the dopamine and serotonin reward systems (Figure 1) This is a notion that is already expressed in different forms and terms, and with different rationales, in Popova et al. [6], as well as Blaber et al. [7], Clément & Ngo-Anh [8], Clément & Reschke [9], and De La Torre [10]. 

Our presentation of the hypothesis works on the foundation laid by these scientists, and takes it several steps further, placing it fully into a context set by evolutionary science, physical anthropology, and the modern social sciences, as well as in a programmatic and socio-political context. Our analysis suggests:

- That space programs must eventually and successfully manage the high level of human neuroplasticity. It is a space program planning issue of utmost importance;
- That human neuroplasticity, at appropriate times, be considered an adaptation that is an advantage for humans in space, if properly managed medically;
- That human neuroplasticity is a foundation for establishing the human species as especially well suited for space, again, with proper medical management;
- That human neuroplasticity enables the human species to eventually join the community of spacefaring species from other star systems, which we hope someday to meet, either tens, hundreds, or thousands of years from now.

Figure 1 in this analysis is particularly useful in illustrating and elucidating the connections between human evolution and human spaceflight, and in stating the hypothesis in more formal, testable terms that can be effectively used by space program planners.

Neurotrophic factor BDNF (brain-derived neurotrophic factor) appears quite stable in spaceflight and may account partly for the continued functionality of human crew. Finally, given the broadly distributed neurological features that are impacted by spaceflight, why are the effects on astronauts not worse? The extraordinary flexibility and versatility of the human species is credited and hope for medical management is sustained in sections following.

### 1.1. Humans in Spaceflight Use Ancient Adaptations

Questions remain regarding long-term spaceflights to Mars, the asteroids, and the moons of the gas giants: which neuroplastic changes are destructive, which can be overcome with time, and which ones need one-time or perhaps continuous medical management? Human neuroplasticity appears, in some ways, to be unharnessed and poorly regulated in a space environment, but our view is that this simply reflects the attempted human adaptation. We shall see in the results that follow that there are changes down to the genetic and hormonal levels in the rodent model in space.

How dangerous are comparable changes for long-term human spaceflight? At this point in time, no one knows for sure. Still, there is knowledge of the comparability in lifetimes among mammalian species. For example, for a rodent whose lifespan is 18 months, one month of spaceflight is equivalent to four years of human spaceflight, i.e., a human with a life expectancy of about 80 years. These comparabilities among mammals of various types provide a solid basis for extrapolating to problems that may be encountered by humans in spaceflight.

The lengthening of the human lifespan during evolution was intertwined with the lengthening of neuroplasticity into maturity. A longer childhood, then adolescence, and finally, a longer adulthood culminating in a longer senescence were all required so that immature humans could be taught by elders, and so that they could teach, in turn. Humans are required to learn and practice complex neurocognitive distinctions and neuromotor tasks, for example, persuasive speech, the intricately patterned cultural rules, rituals, and scientific procedures of their group, and its language, before they can take on the role of “adult”. Adults are now required to manage spacecraft—their maintenance, navigation, piloting, provisioning, and supervision of the crew.

Neuroplasticity allowed all of that learning to occur because human neurology remained flexible and somewhat adaptive even into adulthood. A mature human individual was then able to respond to social cues and others’ needs using neurological pathways that did not yet exist as an infant. This last process now intersects with a process called “the Baldwin effect” which, “by increasing genetic variation or by incorporating evolved peripheral changes to the nervous system” increases the rate of genetic evolution and the level of neuroplasticity [11,12,13]. This is how the theory goes on the *population level*. Here, we focus on ancient, evolved mechanisms that aid individual humans in space.

### 1.2. Humans in Space Rely on Complex Sociality Evolving from 400,000 Years Ago and Longer

The ability of humans to successfully use spacecraft owes much to the complex social learning that occurs over a long lifespan, even in comparison to the ape population from which humans arose. Critical remnants of early hominin evolution continue to benefit adult humans in spaceflight—their agility, flexibility, and neurology remain malleable through adulthood. It is said that they are the most “generalized” species on Earth, and they use that feature to conquer mountain-climbing, spear-throwing, throwing pots, weaving wool, and as we see now, spaceflight. There are downsides to this neurological flexibility in spaceflight. Yet, our view is that there are growing indications that space crews can overcome neurological problems with innate processes plus medical management.

*One of the greatest problems remains and it is programmatic:* which civilians will be able to do the same, and how can we determine who can safely experience space, until spacecraft interiors are fashioned in the distant future to mimic the conditions of gravity, lower radiation, and breathable air on Earth? That is very different from treatment during the long term, upcoming space missions now foreseen. De la Torre and Gonzalez-Torre name the sources of probable failures in crew functioning: “Different risks, both environmental, such as radiation, accidents, and biopsychological, including neurological problems, represent potential sources of subsequent neurocognitive deficits” [14]. From our perspective, the treatment of short-term and long-term spaceflights overlap in planning to become a single over-arching clinical approach, so it is worthwhile noting their principal concerns. 

## 2. Neurological Changes in Spaceflight: Adaptive, Compensatory, or Damaging

Because of the high level of neuroplasticity in humans, neurological changes very likely occur during spaceflight along lines suggested by results on rodents from NASA Neurolab Mission STS90, the International Space Station (ISS), and Russian biosatellites [6], if indeed results are not even more extensive in humans. The average time in space for these samples appears to be about a month, with one flight lasting around three months. These are referred to as “long-term” spaceflights. However, while different species of mammal are not completely comparable, these times in space do not begin to approach the times for anticipated human missions, for example, with crews on the Moon and at the Gateway lunar orbital station, or for spaceflights that venture to Mars. Gathering data will continue to be a challenge for space medicine researchers because they need results for longer times in space. For these researchers and the next generation of astrobiologists using platforms such as the Lunar Orbital Gateway, Elsaesser and colleagues explore a variety of research options, including CubeSats and SmallSats [15].

The reader should keep the length of these flight times in mind, appreciative of the fact that substantial changes were observed by Popova and colleagues over what we interpret as relatively short time frames. The lengths of time over which data were collected also call attention to the scattered reports in the literature of a “bounce-back effect” observed in some studies, and an “overcoming effect” in an analog study where performance difficulties were overcome. Both are discussed below. Questions remain: For humans, would changes be the same, less, or even greater than changes in the rodent model? If the human species is known to have a high level of neuroplasticity, then it should not be surprising that alterations are even greater than in the rodent model. On the other hand, human neuroplasticity may also allow space crew to recover more easily, to the extent that they can. We have named this feature a “dual-valence” human trait [16]. Changes in response to space may be greater, but the capacity to overcome and manage them should also be greater.

Nevertheless, the use of other mammals as models remains valid. Studies provide good indicators of issues to follow up, and fruitful avenues of research for human problems. A variety of changes occur in the central nervous system in spaceflight, but the reasons behind the changes, and the direction of change—good, bad, neutral—are not yet fully demonstrated in the rodent model or among human subjects. Popova and colleagues write: “Long-lasting space travel produced significant changes in genetic control of DA [dopamine] and 5-HT [serotonin] circuits and in neurotrophic factors, but *which of these are adaptive, compensatory or damaging should still be determined*” [6] [emphasis added]. 

### 2.1. Neurological Changes in Specific Genes, Brain Regions, and Neurotrophic Factors

The following are the types of neurological changes found by Popova and colleagues in the rodent model [6].

- During spaceflight, both principal regulators of brain neuroplasticity changed, i.e., neurotransmitters serotonin (5-HT) and dopamine (DA), along with neurotrophic factors (CDNF, GDNF but not BDNF).
- During spaceflight, genetic control of the dopamine and serotonin circuits changed, “especially DA-related genes (TH, MAO A, COMT, D1 receptor, CDNF and GDNF) belong to the risk neurogenes” [6] (p. 396). These are some of the main regulators of neuroplasticity. “Risk neurogenes” are conceived as opposite to “spaceflight-resistant neurogenes” [17].
- During spaceflight, some of the enzymes involved in the dopamine and serotonin metabolic pathways change, but not all enzymes are affected.
- During spaceflight, brain changes are specific to different regions. Popova and colleagues report that the “Substantia nigra, striatum and hypothalamus are highly sensitive to the long-term spaceflight” [6] (p. 396). Again, recall that “long-term” is about one month. These areas of the brain showed reduced dopamine-related and neurotrophic genes. The authors speculate that “Since DA system is involved in the regulation of movement and cognition the data discussed in the review could explain dysfunction of locomotion and behavior of astronauts and direct further investigations to the DA system” [6] (p. 396).

These results appear preliminary, but they begin to suggest features for an etiology of Spaceflight Neuroplastic Syndrome. What is it? Why does it occur? How can we offer the crew some form of remediation for these deep-seated sequelae before artificial gravity in spaceflight is a reality?

### 2.2. Extent, Duration, and Severity of Changes, and Measurement of Them

While the extent and duration of human changes in response to spaceflight are not yet fully known, the number and variety of changes suggest that they appear extensive and very deeply seated. We conclude that their nature and duration will have important implications for all spacefarers, perhaps more for some than for others, until artificial gravity in spacecraft becomes routine and widely available. The possibility exists that additional neurological changes will occur during even longer periods of time in space, without artificial gravity. There is also the possibility that some will lessen in severity with time, and we address that hope below.

Addressing the issue of measuring neuroplasticity will help to clarify all these questions, although, to date, there is no easy and accurate test available [18]. We make several suggestions for measurement later. Direct measures of neuroplasticity may be possible but difficult, for example, tests of changes in neurotrophic factors. Furthermore, there is the issue of how a space program conducts pre-spaceflight tests on, for example, a group of civilian researchers for low Earth orbit (LEO). Proxy indicators of health difficulties may serve as measures for known neurological problems. Thus, clearly one of the next steps is to identify what is changing and then identify (or create) a proxy measure, or several of them.

## 3. A Hierarchy of Methodologies in Operation for This Analysis

It is a tribute to the fine abilities of the human species to observe, record, reason mathematically, reason geometrically, and then create from all of those, hypotheses for testing, and the final accumulation and amalgamation of all these abilities in what, today, we call “theory”. Of course, ancient humans in our species did the same, with growing refinements as they evolved. Their mental manipulations were simply not yet called “method” or “theory”.

The type of fine and difficult work of space neuroscientists, whether using a human model or a non-human model like Rodentia, is now incorporated in stages into the mammoth accomplishment of Charles Darwin (1809–1882) in his theory of the origin of organic species through natural selection [2]. Today, we call this simply “evolution”. Since Darwin’s time, the body of theoretical knowledge has grown enormously, especially in the Extended Evolutionary Synthesis (EES) [3], whose contributions include definition of other mechanisms impacting the origin of species and the fine-tuned micro-changes that occur in populations along the way to the emergence of true species. Indeed, the term “species” has been worked over, critiqued, changed, and questioned over the years. The EES now also includes the mechanisms of evolution at different levels—population level, group level, and individual level—and how those levels interact in the course of the emergence, refinement, and alteration of species at genetic, hormonal, and even cognitive and behavioral levels. The latter have been especially important among the higher Primates and select other species that have some parallel features, such as aspects of the cerebellum of cetaceans and pinnipeds.

### 3.1. “Bench Neuroscience” in an Age of Human Spaceflight

Here, we do not fully recount or critique the fine steps in “bench neuroscience” taken by Popova and colleagues. Instead, the reader is referred to the series of papers on assessments of neurotrophic factors both in space and on Earth and normal and abnormal functioning, published in 2015 [17], 2017 [19], 2019 [20], 2020 [6], 2022 [21], and 2023 [22]. It is noteworthy that the work of these scientists has extended to the application of human disabilities and infirmities.

We acknowledge the difficulty of space neuroscience as a laboratory endeavor. The collection of samples in microgravity is not easy. Keeping animals alive in space so that samples can be drawn is difficult. Correlation of studies from different spaceflights, for different durations, and different reasons is extremely difficult work. Then, the work with samples, verification of original techniques by others, and integration of all those data into a single analytic framework is challenging. Bench neuroscience and all its collection, sampling, and filtration devices, tubes, electrodes, and animal care are very difficult in Earth-based studies, but in spaceflight, it is even harder. Collapsing different studies into a meaningful scientific study design, as in a “meta-analysis”, is very fine, logical, and intuitive work indeed. Nevertheless, humans are good at this, and efforts maximize their intelligence, creativity, and deductive abilities for the benefit of many.

### 3.2. Hypothesis and Incorporation of Theory into the Extended Evolutionary Synthesis

After research data are collected, reviewed, and published, additional steps are taken to incorporate the findings into theory. A step along the way to full incorporation is the hypothesis, and that is the stage presented in this paper on Spaceflight Neuroplastic Syndrome. Additional statistical tests can help to explore the databases on changes, for example, in neurotrophic factors. Ideas are tested, tossed out, and stated in different ways, and, finally, after much thought and manipulation, a hypothesis emerges that needs further testing with new data.

Our hypothesis about the evolutionary origin of Spaceflight Neuroplastic Syndrome is presented within a context in which humans’ high level of neuroplasticity is seen by many as a “disadvantage”, an annoyance, and even a detriment to progress in space programs. This is unfortunate, because, while human neuroplasticity causes problems in human deconditioning in early spaceflights to date, neuroplasticity is also a tremendous advantage. With medical support, it can lead to improvement in human experience in space. Human neuroplasticity is viewed as a “problem to overcome”, but it is more accurately seen as a capacity that grew out of human evolution that *allows* human spaceflight in spite of the problems it causes. These problems can be managed and humans continue to enjoy the status of the best-suited species on Earth to assume its place in space. There is an attitude change to be considered in all this. It is presently preventing the full assumption of humans’ place among spacefaring species (if we find them on exoplanets near or far away). We shall join that community of species eventually, we feel quite sure, even if it is hundreds of years in the future. We are ready for space with human neuroplasticity at a high level. We need to learn to manage it, and with the fine work of Popova and so many other neuroscientists, we shall learn to manage it. We will need it on all those exoplanets we visit.

To summarize, this analysis places human understanding of neuroplasticity at a mid-point of incorporation into the Extended Evolutionary Synthesis. More testing is needed, and a great deal more creative thinking. However, if we fully understand the evolutionary origins of human neuroplasticity, our goal is nearer, our hopes stand steadfast, and we shall enter into an Interstellar Age well-equipped through medical support to manage human deconditioning in spaceflight.

## 4. Hypothesis: Spaceflight Neuroplastic Syndrome, an Attempted Phenotypic Adaptation

Research results on changes in the neurological systems of rats and mice suggest that a type of adaptative reaction is occurring—or being attempted and not fully occurring. The latter may be more accurate because the adaptive response succeeds only partially. Extrapolating to *Homo sapiens* from rodents in spaceflight, we understand that the human species evolved in many different environments all over the African continent, over a lengthy span of time, emerging almost 400,000 years ago [23]. The species has never encountered microgravity before. In many ways, neurological deconditioning in spaceflight can appear as if the human mind and body are attempting a phenotypic response to a new environment—one it hopes to cope with, as it has coped with environments from high-altitudes to frozen wastelands on Earth. However, space is very different from any environment on Earth that shaped the neurological systems under study. *There is a mismatch between selection pressures on the human line and the environment of space, which calls forth an adaptive response that appears to succeed only partly* (Figure 1).

It is important to remember that the selection pressures on the human line occurred over many thousands of years, and the result was the evolution of the dopamine and serotonin systems that humans (and rodents) use today in making selections. When the human body responds to spaceflight, it assumes that there is enough about the new environment that is the same as on Earth. Yet, space is not sufficiently the same, and the dopamine and serotonin reward systems can appear, from observation of a variety of factors, to “malfunction” in comparison to normal performance on Earth. *This causes inaccurate (or at least incomplete) information to be sent to the brain and gut from the two reward systems as they attempt to function in space.* The consequences of this mismatch will be important for program planners to consider (Figure 1).

To the professional observer, the changes in response to spaceflight appear as a biological attempt at a phenotypic adaptation, which usually is understood to not affect the genome (exceptions arise in the Extended Evolutionary Synthesis). However, Spaceflight Neuroplastic Syndrome—as we might call the human version of comparable changes documented by Popova and others—is much more fundamental than that. Spaceflight changes some “neurogenes”—not all genes supporting the human neurological system, but some. Others remain stable.

*The genes that change make human neural deconditioning in spaceflight more than a phenotypic adaptation*. It is not an adaptation within a range that the species has encountered before. Phenotypic changes are common, and they do not fundamentally disrupt basic processes or the genome. Spaceflight is another matter. It disrupts the metabolism of the very important dopamine and serotonin pathways, which make the species “successful” and stable from an evolutionary perspective through the choices it has made and continues to make.

Therefore, *it is very interesting that human reactions to space have not been worse.* One reason that outcomes may not be as bad as would seem congruent with the species is that they may sometimes begin to reverse. This has been observed by Hupfeld and colleagues in human behavioral performance, directly after spaceflight. They have named the human pattern that follows experience in space first, by dysfunction, and second by adaptive plasticity, as SPACeD, or “Spaceflight Perturbation Adaptation Coupled with Dysfunction” [24].

### 4.1. Spaceflight Findings to Date in a Positive Evolutionary Perspective

At the present time, there is a great deal of interest in documenting the human neurological problems that emerge in spaceflight. Iwase and colleagues summarize the “Effects of Microgravity on Human Physiology”, including some neurological sequelae [25]. Neurovestibular system changes have been among the best described, partly because they are so obvious, uncomfortable, and ubiquitous among astronauts. Humans are Earth-evolved Primates whose hearing and balance capacities, which are quite substantial, attempt to adapt to a gravity-less environment. The results appear, like many of Popova and colleagues’ findings on neurological changes in spaceflight [6], to be serious efforts at phenotypic adaptation so that humans can cope with weightlessness. This is not required on Earth and has never been required of any species on the Primate lineage from which modern humans are descended. Microgravity is not just “new”. It causes body systems to flail in the face of overwhelming changes to which they cannot phenotypically adapt. We interpret the variety and number of changes that Popova and colleagues describe as an inability to organize a combined effort to adapt phenotypically.

There is a mismatch between the evolved capacities of the human neurovestibular system, and what is asked of it in an entirely new environment. We marvel at how human systems manage to function as well as they have in spaceflight. It is a testament to human flexibility, agility, plasticity, and especially neuroplasticity. This is a perspective contrary to many researchers who, understandably, remain very concerned, as we do, for the safety of humans in space. There has been little room for respect for just how good the human species’ attempts have been to adapt to microgravity.

We hold that an appreciation of human capacities in an evolutionary context will allow researchers and space physicians greater latitude in devising solutions that help human workers adjust to space and accomplish the tasks that lie ahead of them: serving as crew on roundtrips from Earth to Mars; building giant spacecraft in lunar orbit that can support teams on long voyages to the asteroids and outer planets; and surviving on the lunar surface with exercise, diet, and medication management so they can build the administration and spaceflight center that Earth’s Moon is destined to become for the Solar System.

An appreciative perspective relies on knowledge of how evolution operates. Iwase and colleagues describe one human system after another that attempts to compensate for the absence of gravity [25]. What is so remarkable is not how much these systems have failed, but how much they have succeeded in adapting to an environment that Primate evolution was never meant to encounter.

### 4.2. Stable Components of an Attempted Phenotypic Adaptation to Spaceflight

It is an important question for future researchers to ask why some neurotrophic factors in rodents changed in space, but BDNF (brain-derived neurotrophic factor) did not [6]. BDNF is the only neurotrophic factor considered in that research that did not change, and it supports the serotonin system. Serotonin is largely (95 percent) stored in the gut. It regulates mood, cognition, reward, memory, sleep, and stress. The stability of BDNF in the rodent model could be seen as a useful clue if it carries through to humans. Below, we discuss medical research findings that suggest there is a good chance it does.

It should also be noted that the stability of BDNF gene expression described by the Popova team is to an extent a reflection of the time span between mice landing and animal sacrifice. This may indeed be enough time to mimic spaceflight-induced changes in BDNF gene expression. This hands-on research situation highlights the necessity of immediate data collection in space neuroscience. Time is of the essence when gravities are changing. The collection of data on mammals is a critical chore to provide the least alteration to changes upon landing in full gravity.

Another important question is why some of the enzymes in the metabolism of dopamine and serotonin in the rodent model change in response to spaceflight, but others do not. Again, in humans, this may point toward some kind of medication management.

A final important question is why some parts of the brain are highly sensitive to spaceflight, but others are not. This difference reminds us that throughout the evolution of the human brain, changes occurred in different places, at different times, and different rates, especially features involved in the increasing lateralization of the brain [26,27].

Lateralization is often involved in the emergence of new skills and advanced neuro-cognitive traits on the evolutionary line leading to *Homo sapiens* because it often involves the exaptation of tissues on one side of the brain that were previously used for other functions—thus creating asymmetry [5]. Both lateralization and asymmetry are marked features of the “hominine clade”, and according to Gómez-Robles, Hopkins, and Sherwood, these features render the brain of humans and near relatives “highly evolvable and responsive to selective pressures” [28] (p. 1). These authors also report the following, and in so doing, link plasticity and asymmetry in both brain evolution and corresponding cognitive evolution:

Our results emphasize two key properties of brain evolution in the hominine clade: first, evolution of chimpanzee and human brains (and probably their last common ancestor and related species) is not strongly morphologically constrained, thus making their brains highly evolvable and responsive to selective pressures; second, chimpanzee and, especially, human brains show high levels of fluctuating asymmetry indicative of pronounced developmental plasticity. We infer that these two characteristics can have a role in human cognitive evolution [28].

We conclude that this group of Primates gained great advantages from plasticity, and so, there were extremely good reasons for the retention of a high level of plasticity, and even reasons for it to increase, which Axelrod and colleagues explored recently in the essay, “Integrating Neuroplasticity and Evolution” [13].

It appears to us that lines of thought are converging that neuroplasticity is an inherited advantage for the hominins (humans, chimpanzees, and gorillas), and while it is now causing problems at the beginning of humanity’s adventure in space, it will be extremely useful while the asteroids, outer planets, and new exoplanets are explored. The species will retain and make use of its substantial neuroplasticity, allowing it a future in space.

### 4.3. Stability of BDNF and Human Mental Health in Spaceflight

Returning to research on neurotrophic factors, if BDNF is indeed quite stable for mammals during spaceflight, as research on rodents suggests, then the functions it supports may be stable, as well. Therefore, let us examine some of the documented effects of the opposite: lower levels of BDNF, i.e., when it is in a deficient state. These effects could point to the types of stability BDNF imparts in spaceflight.

There is growing evidence that lowered BDNF is associated with psychiatric disorders: “Dysregulation in 5-HT–BDNF [serotonin-BDNF] interaction may be responsible for development of neuropsychiatric and behavioral abnormalities” [20]. It is also true that BDNF can serve as a kind of medication. The “neurotrophin hypothesis of depression” has been confirmed: “Robust empirical findings indicate an association between increased BDNF gene expression and peripheral concentration with improved neuronal plasticity and neurogenesis” [29]. The conclusion we draw is that deficient BDNF is the root of some pathological human conditions and alleviating a low level creates improvement.

It appears reasonable to assume that a stable BDNF imparts psychological stability in spaceflight, and so to test its adequacy seems important. Popova and Naumenko describe “the prolonged positive effect of BDNF on genetically and epigenetically defined central nervous system disorders” [20] (p. 227).

More recently, there have been findings that disorders involving neuroplasticity are important in autism spectrum disorders [22]. Depression, neuronal loss, and cortical atrophy appear to be correlated with lowered levels of BDNF. The resumption of normal levels of BDNF is linked to antidepressants, according to Martinowich and colleagues [30]. The neurotrophin hypothesis of depression is based heavily on the correlation between lower levels of BDNF and a higher frequency of depression, depressive symptomatology, neuronal loss, and cortical atrophy; restoration of BDNF is linked to the administration of antidepressants, according to the same researchers. While Popova and colleagues’ results are for rodents [6], it is difficult to avoid the conclusion that the stability of BDNF in spaceflight could be interpreted as a positive feature for humans, given previous clinical results.

Furthermore, if BDNF has been assayed for purposes of research on depression and anti-depressants, then a test could be readily available to evaluate non-astronaut applicants for spaceflight. Such an assay might serve as a suitable measure for the evaluation of larger numbers of people for experience in space when facilities are available. On the other hand, if BDNF is as stable in humans as in rodents, there may not be much information gained. The question remains: Is it? Further testing would indicate whether it is a distinguishing measure useful as a qualifier for civilian spaceflight. It will be a matter of research to confirm that BDNF imparts neurological stability for humans in spaceflight, and if so, whether it varies sufficiently to be predictive for non-astronaut applicants.

We hasten to add that BDNF is not the only component in the functioning of dopamine and serotonin systems to be involved in aggression, depression, and suicide, and psychiatric disorders. Popova and colleagues address the different kinds of serotonin receptors and the “dysregulation in central serotonergic (5-HT) neurotransmission” [21]. Clearly, the unraveling of the biochemistry of mental health is complex. However, BDNF appears to offer hope as at least one source of human stability in spaceflight, and perhaps an avenue toward a type of assay for larger numbers of spacefarers who will soon want to experience spaceflight.

### 4.4. Astronaut Decision Making Remains Effective

There are many reasons why Spaceflight Neuroplastic Syndrome is important, not least of which is that the human serotonin and dopamine systems are involved in making choices and decision making. It appears that one cannot assume “all is well” when spaceflight disrupts the basic processes at the foundation of human decisions. The still unanswered question is: How bad is such a syndrome for humans in space? To date, one of the best available workarounds appears to be group decision making [31], including Mission Control staff.

Logically, we arrive again at the same type of question: Why are reports on human decision making in space not worse, *if* space disrupts quite a variety of genes, neurotrophic factors, and enzymes related to it? Perhaps there are enough major elements of the human dopamine and serotonins systems that remain stable, so that, quite often, normal functioning prevails, or it recovers quickly as spaceflight lengthens in time.

Another explanation may be that, because human decision making is extraordinarily complex, any questionable decisions are corrected by other crew, by Mission Control, by an AI, or by the self. At first, it might appear that this would take a very long time but let us recall that human decision making, even among several people, can occur at lightning speed in a crisis. We provided a narrative of what decision making in space “feels like” elsewhere [16]. It is taken from a former astronaut’s diary. We see in that excerpt that astronaut decision making in space is remarkably quiet, tense, soft-spoken, yet ready for action, and extraordinarily fast when it occurs. In a cultural manner, this mimics the demeanor of so many test pilots who were among the very first astronauts. They were trained to remain very calm and clear thinking in a crisis, and we have been thankful for that training more than once.

The human species has often encountered heightened CO_2_ levels, as in forest fires, but not on a constant basis. Some radiation is a natural part of life on Earth, and all animals adapt to some degree to it. Still, no species is accustomed to the levels of cosmic radiation encountered in space, with atoms whose electrons have been torn away while they travel at very high speeds, plus high-energy protons and heavy ions from outside our solar system [32]. If we had to prioritize problem solving for the three main environmental problems in spaceflight, weightlessness would surely be first, radiation second, and CO_2_ third unless the latter were to reach deadly levels, which it can.

### 4.5. The Hanging Question from Research: Why Are Sequelae Not Worse?

Given research results to date, we are left with an unanswered question: Why are humans in space apparently not experiencing more problems? Why are the sequelae not worse? We did not find the question stated in the literature in quite this way, although we understand that reports are very recent on research findings with as broad a variety of neurological changes as Popova and colleagues report [6,19].

We acknowledge that issues related to neurovestibular problems can be severe and impact health and work routines. Problems with locomotion can be major issues, both in space and upon return to Earth. We are not minimizing problems and we acknowledge that findings can appear substantial, for example, in brain connectometry [33]. However, the depth and variety of changed neurological features identified by Popova and colleagues [6] suggest that problems among the more than 600 people who have been in space would have been greater in number and severity. The human species’ flexibility and ability to overcome changes in space appear to be remarkable.

There is a possible explanation for the level of neurological problems, besides the fact that astronauts are usually in prime physical and mental condition. It may be that there are few tests and indicators sufficiently sensitive, to date, to detect human decision-making problems and other higher-level cognitive faults. Some cognitive tests have been administered. For example, Basner and colleagues report on the use of a cognition test battery for spaceflight, and they initially acknowledge that “…the nature of neurobehavioral functioning in space has not been clarified” [34]. The NASA Task Load Index has been administered to measure mental demand, physical demand, temporal demand, performance, effort, and frustration [35,36]. Use of the latter has apparently been quite extensive, and useful. For a recent review of neurocognitive assessment in microgravity, see De la Torre and Gonzalez-Torre [14].

## 5. Hypothesis: Spaceflight Neuroplastic Syndrome May Decrease with Time and Treatment

### 5.1. Research Priorities to Support the Success of Future Space Programs

It is important to keep in mind that there is no firm evidence yet of a connection between the deeply seated neurological factors identified by Popova et al., and others, and oft-used cognitive test batteries. There is good work connecting low BDNF with depressive and other psychiatric disorders on Earth—even borderline personality disorder [37]—but no assessment of the effects on cognition in space of the changes in neurotrophic factors, enzymes, and genes at risk in space. That may require proxy measurements and new test batteries. It will surely require test periods longer than a month.

Consequently, without firm evidence, it becomes difficult now to target medication management for the entire complex of neurological changes—the Spaceflight Neuroplastic Syndrome. Is medication management targeted at neurotrophic factors? Or, at the metabolism of serotonin or dopamine? Or, at the genetic level, at “risk neurogenes”? Finally, we ask whether the best choice is to do nothing, and encourage exercise and diet? With good health and training, humans appear to be able to tolerate space, for a time, without much medication management. Again, data are needed on longer missions to determine if neurological changes persist, increase, or reverse. Given the nature of human neuroplasticity, they could reverse somewhat and stabilize.

These possibilities need close examination, especially if they are based on therapeutic models for the medication of non-spaceflight problems. At present, Spaceflight Neuroplastic Syndrome is not sufficiently well characterized in terms of overt symptomatology that it can be measured easily. In the next section, we see a model that attempts to connect neurological changes and behavior.

### 5.2. Managed Bounce-Back with an Earlier Timing for Recovery

Hupfeld and colleagues describe a sequential pattern during human spaceflight of dysfunction followed by “adaptive plasticity” [24]. They propose a model of “recovery” post flight. Their proposed stages take place within a full social and psychological context, including background and resilience factors.

Each stage involves behavior, brain structure, and brain function, comprising a conceptual structure that would, if followed, begin to connect brain changes, cognition, and behavior. Of special importance is a reference to a decline in the performance of a dual task by astronauts, followed by recovery post spaceflight.

It would be very useful—with a careful understanding of the indicators of “recovery”—to initiate this recovery stage earlier, even during spaceflight. This approach would aid post-flight recovery, or it could even allow a lengthened time in space with improved functionality. A managed bounce-back is a concept that needs exploration.

The authors’ use of the term “adaptation” is different from its use in evolutionary science. In the context of evolution, adaptation has a positive connotation, leading to a “better adaptation” to an earthly environment—either affecting the genome (genotypic) or not (phenotypic). In space, a mammal’s attempt at a phenotypic adaptation (or even genotypic, following Popova et al.’s findings on gene changes) causes various kinds of problems, and so it appears “maladaptive” either during spaceflight or back on Earth. This is captured by Hupfeld et al.’s term “recovery”, implying that adaptation has caused health problems. That is very different from its use in evolutionary science.

This confoundment is only unraveled by understanding that an adaptation in microgravity may have unhealthful consequences. The literature has yet to clarify this issue and to simply use a different term. Clearly “adaptation” is always relative to environmental context. We solved this issue here by using the term “attempted adaptation”. Their use of “dysfunction” implies the same, i.e., an attempted adaptation that fails, perhaps partially (as we see in Popova et al.’s findings). A true phenotypic adaptation would remove a mammal’s problems, not cause them.

### 5.3. The Overcoming Effect

“Recovery” implies “recovery to normal”, as in a healthy state on Earth. However, that may not be possible for crew in spaceflight. There are multiple factors operating in addition to microgravity, including heightened CO_2_ and cosmic radiation. Recovery to functional states where tasks can be accomplished and decisions can be made may be the realistic goal. If crew are trained to anticipate a pattern of decline in functionality, they may be likely to attempt to counter it. Scully and colleagues’ findings among “astronaut-like subjects” strongly suggest that “overcoming” some of the sequelae of spaceflight may depend on training, physical and mental condition [38], and we would suggest, as well, agency and will in overcoming the effects of spaceflight. All of us have experience with such an effort, for example, an unexpectedly fast roller coaster ride, a whiff of propane gas that causes momentary dizziness, or the like. We all know the experience of attempting to set ourselves right and focus on the tasks at hand. The human species is particularly adept at this, especially when aided by other trained members of the crew, or Mission Control, whose staff become sociologically “members of the group”.

### 5.4. Human “Focused Activity” to Compensate in Spaceflight

The notion of an “effort to overcome” finds some support in the results from Satish and colleagues. At heightened CO_2_ concentrations, large and statistically significant reductions occurred on seven of the following scales of decision-making performance, *but not “focused activity”*: (1) basic activity level; (2) applied (opportunistic) activity; (3) focused activity; (4) task orientation; (5) initiative (new activities); (6) openness to information search; (7) information usage; (8) breadth of approach (flexibility); and (9) basic strategy (number of strategic actions). “Focused activity” comprises “strategic actions in a narrow endeavor” [39].

The results on “focused activity” as a type of cognitive action state run contrary to results on the other cognitive measures.

Our interpretation can only be stated within the context of other results on human performance in spaceflight. The measure “focused activity” appears to imply intensity, effort, agency, and will. While it is not a precise measure of the will to overcome spaceflight sequelae, we understand focused activity as broadly indicative of a self-conscious effort to prevail and self-correct. It cannot be dismissed that the self-aware species that the human represents can well focus on his or her own health as a crew member and make added effort to perform. The human focus, effort, will, and agency—whatever it is named—may extend to the overcoming of the physical sequelae of spaceflight, to an extent. It cannot be dismissed, not with the results of Satish and colleagues.

### 5.5. The Holdover Effect: Is It Tolerance?

Findings on the brain’s perivascular spaces (PVSs) show novice astronauts had increased total PVS volume, pre to post flight, but experienced crew members did not (*p* = 0.020) [40]. Experienced crew appears to have “holdover effects” from prior flight(s) that protect them from certain changes. We would ask whether a type of “tolerance” for spaceflight emerges among astronauts, if tolerance is defined as an ability to withstand continued exposure to a drug or an environmental condition without a negative reaction. Could tolerance for spaceflight emerge among civilian researchers who spend several months, or more, in LEO, just as it appears to emerge among astronaut crew, to an extent? It is a notion worth exploring.

### 5.6. Mitigations Using Artificial Gravity

The prolonged microgravity of space produces significant physiological deconditioning that, among other things, affects cognitive performance [41]. Various mitigations have been investigated, among which is an obvious emphasis on adequate diet, sleep, and exercise. However, the most intuitive countermeasure is to supply the spacefarer with continuous or intermittent artificial gravity. Early results from Blue and colleagues [42] report no significant adverse physiological responses to G forces generated by experience in a centrifuge, even by subjects who were recruited based on five disease categories (cardiovascular disease, diabetes, lung disease, back or neck problems), and there was a control group.

Later methodologies, including Earth-based simulations of microgravity, particularly exposure to head-down-tilt bed rest (HDBR), have shown changes in the functional connectivity in the brain network in a relatively short time—three days [43]. However, this study joins that of Rabineau and colleagues [44] in having somewhat inconclusive results when the test subjects are exposed daily for 30 min to artificial gravity.

The artificial gravity results from mice on the International Space Station have been more encouraging [45]. When the mice were continuously exposed to 1× *g* in centrifugal cages, the atrophy of their thymus, in comparison to a control group of mice experiencing microgravity, was reduced by about half. This significant mitigation due to artificial gravity bodes well for the human immune system [46]. The challenge now is to implement artificial gravity for humans in spacecraft. It can be achieved by the steady acceleration of the spacecraft, followed by de-acceleration as it approaches its target, or by rotation of all or part of the spacecraft [41]. Solutions have been contemplated for a half-century or more, and the time is coming to test them in space on human crew.

### 5.7. Mitigation Issues in Planning for Civilian Spacefarers

The likely solution to “recovery” (whether post spaceflight or while humans remain in space and begin a process of normalizing for Earth’s environment once more) will almost surely *combine human neurological capabilities imparted by a high level of human neuroplasticity, and medical remediation of various types*, perhaps different regimens for different people. The latter is a notion that Hupfeld and colleagues also suggest about post-spaceflight recovery. As they detail, it depends on factors identified in the categories of life course experience, stressors, and resilience [24].

It is difficult to emphasize too much how important adequate regimens will be when larger numbers of civilians, researchers, and construction workers begin to populate low-Earth orbit (LEO) and experience space. Assessment tools will be needed to quantify their likely difficulties in a new space environment and to evaluate them regularly during their time off Earth. Their lives may depend on it.

## 6. Toward an Etiology of Spaceflight Neuroplastic Syndrome

The impression from changes documented by the Popova et al. and Hupfeld et al. research teams, and others, is that, while deeply seated neurological changes appear somewhat scattered, they are also substantial, as if the mammal has encountered something fundamentally new and is trying to adapt as well as possible. Popova and colleagues have documented quite a few changes in just a month’s time in spaceflight, in the rodent model, and while the duration of the neurological changes is not yet certain, one is left, again, with a rather contrary question:

*If there were this many changes in the rodent model, why have the externally observed effects we know from humans in space (who have greater neuroplasticity) to date, not been worse*? Why are the effects found in rodents so seemingly scattered? The most elegant answer is that the species is attempting to adapt, succeeding in some ways, but failing to do so, in other ways. Can this understanding be extended to humans?

One obvious but rather circular answer to the question is that humans have a high level of neuroplasticity, i.e., they change neurologically a lot, and rather easily. They bounce back equally easily, sometimes. Rodents appear to have some, but not as much neuroplasticity. Yet, when we began our research, we did not realize the diversity of neurological changes that would occur when an environment like space is encountered. The fact that over 600 human beings, to date, have encountered space for various time periods and have not been completely overwhelmed appears to us to be an interesting observation. The case of astronaut John Glenn is à propos. He went to space twice, once as a young man, when he orbited the Earth in 1962, and much later, in 1998 when he was 77 years old, he went on a nine-day mission [47].

We recall watching the broadcast the day of that second flight, and it took some time for him to recover and walk. Still, at that age, he was able to come back and function. This underscores that humans, even at 77 years old, maintain an extraordinary level of agility and ability to adapt. Many observers say that humans are less able to adapt as they get older, but we wonder just how true that will be on a population basis, when spaceflight is encountered, and when diseases of senescence will cause older people to seek relief in microgravity.

The task before us is to apply what we have learned from the human experiences of over 600 people in space, along with other mammals who reflect much, but not all, of our own biology. Many more people are set to enter space soon, including researchers, managers, and administrators while NASA-supported facilities are turned over to civilian hands and many more become involved in environmental monitoring of Earth from orbit.

What we cannot lose sight of is that humans are a product of a remarkable evolutionary line of Primates. Spacefarers capture and will use all their evolved talents and add to them human insight, sensitivity, agility, and ability to problem-solve in groups. The latter is especially unique to the human species [31], and requires further research to substantiate exactly how group problem solving can be maximized in space. Neuroplasticity was inherited from a worthy line and then augmented among the hominins. It is causing problems, but we see signs in the current literature that these problems can be remediated with both traditional and very modern health care and medical management.
